# Avian roosting behavior influences vector-host interactions for West Nile virus hosts

**DOI:** 10.1186/1756-3305-7-399

**Published:** 2014-08-28

**Authors:** William M Janousek, Peter P Marra, A Marm Kilpatrick

**Affiliations:** Department of Ecology and Evolutionary Biology, University of California, Santa Cruz, California 95064 USA; Migratory Bird Center, Smithsonian Conservation Biology Institute, National Zoological Park, Washington, DC 20008 USA; Avian Science Center, Department of Ecosystem and Conservation Sciences, University of Montana, Missoula, Montana 59802 USA

**Keywords:** Sociality, Group size, Vector-host contact rates, Vector:host ratio, Model, Flocking, Fitness, Evolution

## Abstract

**Background:**

Extensive work has shown that vectors almost never feed at random. Often, a subset of individual hosts and host species are fed on much more frequently than expected from their abundance and this can amplify pathogen transmission. However, the drivers of variation in contact patterns between vectors and their hosts are not well understood, even in relatively well-studied systems such as West Nile virus (WNV).

**Methods:**

We compared roosting height and roost aggregation size of seven avian host species of WNV with patterns of host-seeking mosquito (*Culex pipiens*) abundance at communal and non-communal roost sites.

**Results:**

First, host-seeking mosquito abundance increased with height and paralleled increased mosquito feeding preferences on species roosting higher in the tree canopy. Second, there were several hundred-fold fewer mosquitoes per bird trapped at American robin (*Turdus migratorius*) communal roosts compared to non-communal roost sites, which could reduce transmission from and to this key amplifying host species. Third, seasonal changes in communal roost formation may partly explain observed seasonal changes in mosquito feeding patterns, including a decrease in feeding on communal roosting robins.

**Conclusions:**

These results illustrate how variation in habitat use by hosts and vectors and social aggregation by hosts influence vector-host interactions and link the behavioral ecology of birds and the transmission of vector-borne diseases to humans.

## Background

The behavioral ecology of hosts can play a significant role in determining pathogen transmission dynamics. For diseases where transmission is direct, host foraging ecology, habitat preference, and social interactions, including mating strategy, can influence the probability of contact with an infected host or environment and create hotspots for transmission [1–4]. However, the impact of animal behavior on the transmission of vector-borne pathogens is less clear [5, 6]. Most studies exploring the influence of host behavior on vector contact rates have examined how human behavior influences the transmission of pathogens causing malaria, dengue fever, and Chagas disease [7–9]. Understanding and controlling infectious diseases that infect wild animals (many of which infect multiple species, including humans) requires examining the behaviors that influence contact between vectors and hosts [10–13].

An ideal free distribution model developed for host-seeking vectors predicts that vectors will feed on hosts to maximize their feeding success, and the degree of heterogeneity among and within species will vary with host and vector densities, and host defensive behavior [14]. However, the importance of these factors, as well as host habitat use and aggregation in determining the extent to which vectors successfully move across the landscape to maximize feeding success is unknown.

Although animals are well known to form communal groups to reduce the risk of predation [15], it is less clear whether aggregations form in response to biting arthropods and the pathogens these arthropods carry [16]. For example, although herd formation in caribou reduced biting for individuals in the center of a herd, the temporal pattern of formation of groups suggested aggregation was more for thermoregulatory reasons than in response to biting flies [17, 18]. Similarly, while parasitism has been shown to increase with sociality in some birds [19, 20], in other taxa infection intensity of endo- and ectoparasites often decreases with increasing host group size [21]. At least as important as the influence of biting arthropods on the formation of communal groups is the reciprocal impact of host habitat use and group formation on the transmission of vector-borne diseases such as West Nile virus (WNV).

West Nile virus was first detected in North America in 1999, and is primarily maintained in an enzootic cycle between avian hosts and mosquito vectors [11]. Nocturnal-feeding mosquitoes of the genus *Culex*, specifically *Cx. pipiens*, *Cx. restuans*, and *Cx. tarsalis* are the most important enzootic and bridge vectors of WNV in urban, residential, and agricultural areas in North America [6, 22–25]. Although over 200 species of birds have been found to be infected with WNV, mounting evidence from the mid-Atlantic, Northeast and Midwestern USA, and Colorado suggest that a single species, American robin (*Turdus migratorius*), is likely responsible for infecting the majority of WNV-infectious mosquitoes [26–31]. However, the mechanisms leading to preferential mosquito feeding on this species, and avoidance of others are unknown.

One potentially important and largely unexplored aspect of WNV transmission ecology is the behavior of hosts, including their nocturnal roosting patterns, which determine their location during the feeding periods of the dominant WNV vectors. Although host defensive behavior [32–34] and traits such as age [12, 35] have been shown to influence the success of feeding mosquitoes, the influence of roosting behavior has rarely been explored. Two aspects of nocturnal roosting behavior are likely to influence contact rates with foraging mosquitoes. First, host-seeking *Culex* mosquito abundance may be higher in the forest canopy [36–39] so birds roosting higher may be exposed to more mosquito bites. Second, the formation of communal roosts could influence vector-host contact rates in several ways. Multiple birds produce more CO_2_ than an individual bird, which could increase the number of mosquitoes attracted to the group. However, the increase in host seeking mosquitoes may not be proportional to group size, in which case communal roosting would decrease the average number of mosquitoes feeding on each bird. Previous research at communal roosts of American robins in Connecticut found fewer numbers of *Culex* mosquitoes were trapped at roosts versus non-roost sites, but WNV prevalence in mosquitoes was higher at communal roosts [40], whereas in Illinois there were no significant associations between distance to an American robin communal roost and WNV-infection in mosquitoes [41]. However, neither of these studies determined the number of host-seeking mosquitoes per bird at communal roosts and non-roost sites. The per capita number of vectors per host (i.e. vector-host ratio) is an important factor for vector-borne transmission and disease exposure [14, 42, 43].

We studied roosting height and roost aggregation size of seven bird species, and simultaneously quantified mosquito host-seeking abundance at the same sites. The seven species include the most important amplification host for WNV across a wide range of North America, the American robin [26–30, 44], and the most abundant bird species in many urbanized and agricultural areas of North America, house sparrows and European starlings. We hypothesized that avian species roosting higher in the canopy and in smaller groups would have higher per capita contact rates with host-seeking mosquitoes and might explain preferential feeding by mosquitoes on those species [27, 28, 44]. We further hypothesized that mosquito abundance would be larger at communal roosts of birds, but not proportionate to roost size, such that the per capita number of mosquitoes would be smaller. In addition, because large groups of birds emitting CO_2_ could diminish the trapping efficiency of mosquito traps (which also use CO_2_ as the primary attractant), resulting in biased estimates of mosquito abundance at communal roosts, we performed an experiment with artificial sources of CO_2_ to estimate the magnitude of this effect.

## Methods

Our study was conducted at three sites in Maryland and Washington, DC [25] including an urban area in Foggy Bottom, DC (38° 54’ 07.33” N, 77° 3’ 18.27” W), a residential area in Takoma Park, MD (38° 58’ 23.55” N, 77° 0’ 18.80” W), and a predominantly forested area in Rockville, MD (39° 6’ 44.88” N, 77° 6’ 23.80” W). These field sites were part of an ongoing multi-year study on WNV transmission in the greater Washington, DC area where data on mosquito feeding patterns, host abundance, and feeding preferences have been previously collected [28, 29]. Data on avian nocturnal roosting behavior and host-seeking mosquito abundance were collected from July through September 2010, when WNV infection prevalence peaks in mosquitoes and when most human infections occur in the eastern USA [29, 45].

### Roosting behavior

We attached transmitters (Lotek Wireless Inc. Newmarket, Ontario) weighing less than 5% of an individual’s body mass [46] to seven bird species: American robin (transmitter weight: 2.8 g, N = 89 individual birds), Northern cardinal (*Cardinalis cardinalis*, 1.5 g, N = 60), tufted titmouse (*Baeolophus bicolor*, 0.7 g, N = 5), gray catbird (*Dumetella carolinensis*, 1.4 g, N = 15), mourning dove (*Zenaida macroura*, 2.8 g, N = 8), European starling (*Sturnus vulgaris*, 2.8 g, N = 7), and house sparrow (0.7 g, N = 29). All birds except one American robin and six of the European starlings were juvenile (hatch-year) individuals. We focused on juvenile birds for the roosting studies because they appear to play a key role in the transmission of WNV [47]. Birds were caught by removing nestlings from nests found by observing parental behavior (American robins and Northern cardinals), or were captured using mist-netting (all other species). Birds were then banded with an aluminum USFWS and unique color band combination, radio-tagged, and released. All research with birds was approved by animal use and care protocol Kilpm1112 from the University of California Santa Cruz’s Animal Care and Use Committee.

We attached transmitters using a figure eight shaped two-loop harness design that slid over the legs of the bird and allowed the transmitter to rest on the synsacrum [48]. Harness loops were made from clothing elastic, which has been shown to work as a durable but less-constrictive material for short-term attachments of transmitters to birds [49]. Harness size was adjusted to individual body size and fixed with super glue to prevent unraveling. We observed post-banding movements of tagged individuals to insure attached transmitters were fitted properly and that nestlings continued to be fed by parents.

We used Biotracker scanning receivers in combination and three-element folding yagi antennas (Lotek Wireless Inc. Newmarket, Ontario) to locate birds fitted with transmitters. To record nightly roosting location of tagged birds, bearings were taken simultaneously from two or more points for triangulation. Once the approximate location of a bird was discovered we used ToughCam thermal imaging cameras (Infrared Cameras Inc. Beaumont, TX, USA) in tandem with radio receivers to determine the roosting location and height of individuals [50]. We estimated the height of the bird using a clinometer, a 50 m tape, and the bird’s location.

Over the course of the study we found several tagged individuals roosting in large communal aggregations while others remained solitary. We estimated communal roost aggregation size by counting the number of birds arriving from the four cardinal directions at dusk and by counting all birds within the contiguous vegetation area surrounding the roost with thermal imaging cameras. Roost size estimation methods were used concurrently and validated against one another to ensure both methods produced similar results. The two American robin communal roost sites were 7,128 and 2,380 m^3^ in size and at peak occupation contained roughly 2.5 and 4.1 American robins per m^3^ respectively. When a bird was found roosting solitarily, not in association with a large communal aggregation, we searched within a ~10 m radius of the bird using a thermal imaging camera to determine if additional hosts were roosting in close proximity. Previous research has shown that 10 m is the approximate distance that foraging mosquitoes respond to hosts [51].

### Host-seeking mosquito abundance

We measured the abundance of host-seeking *Cx. pipiens* mosquitoes, at four sites (two non-communal and two communal roost sites) in Takoma Park, MD using two to six CDC light traps hung at two heights: 1.5 and 12 meters, and baited with CO_2_ (~300 g dry ice per trap). We identified mosquitoes using morphological characteristics [52] and identified a subset of *Culex* mosquitoes using a molecular assay to determine the fraction of *Culex* mosquitoes that were *Cx. pipiens* [53]. We were unable to test mosquitoes trapped at these sites for WNV because samples were lost in shipment to the testing lab.

Communal roost sites consisted of large American robin aggregations discovered while radio-tracking robins. Mosquito traps at communal roost sites were placed within and on the edge of roosts. Non-communal roost sites were the location where birds were initially banded. All four sites (two communal and two non-communal) were within a circular area with an approximate radius of 10 km and consisted of relatively similar residential areas with small (e.g. <2 Ha) green spaces along stream corridors.

We assessed variation in host-seeking abundance of mosquitoes along a vertical gradient at four trapping locations in Foggy Bottom, DC between July and August 2010. We hung single trap-lines consisting of three CDC light traps baited with CO_2_ at varying heights: 1.5, 6, and 10–15 meters with the highest trap height dependent on tree height.

### Roosting height and mosquito feeding preferences

We examined how roosting height might influence mosquito feeding preferences by using mosquito bloodmeal analysis data from a previous field study [28] conducted at the same field sites, and compared those data with the roosting height for the species in our study. In the previous study, the preference for a species was estimated as the fraction of blood meals from a species divided by its relative abundance, estimated using four to six unlimited radius point counts at each site conducted monthly [28]. We limited our analysis to the average roosting height of juvenile birds, which appeared to differ in roosting height from adults although sample sizes prevented a rigorous statistical analysis. Thus, our analysis was restricted to five (American robin, Northern cardinal, Gray catbird, Mourning dove, and House sparrow) of the seven species in our roosting study. We had insufficient data to include European starlings in this analysis, and Tufted titmice were not present at the two sites where we had sufficient numbers of blooded mosquitoes to estimate mosquito feeding preferences.

### Simulated roost experiment

We attempted to test the hypothesis that avian hosts roosting near a mosquito trap decrease the number of host-seeking mosquitoes caught in a trap. We compared mosquitoes caught in CDC light traps baited with a single 0.66 Gallon Igloo cooler full of dry ice with traps surrounded by four additional dry ice filled coolers spaced evenly around the trap in a spiral staircase pattern at a distance of 0.5 – 1.0 m from the light trap with one in each cardinal direction. We conducted this experiment at Foggy Bottom, DC and Takoma Park, MD. The experimental design was chosen to mimic the host densities observed around mosquito traps at actual communal roost sites. We estimated that the four additional coolers produced the same amount of CO_2_ per hour as 372.4 ± 24.8 additional hosts, based on estimated field metabolic rate of American robins [54]. We used field metabolic rate rather than basal metabolic rate because many birds at communal roosts were moderately active during the night, including vocalizing and flying between perches. We ran traps for one night as described and then alternated the location of the additional coolers of dry ice. We replicated this experiment across four trapping locations and eight nights for a total of 32 trap nights.

### Correcting mosquito abundance estimates at communal roosts

We attempted to correct for the influence nearby hosts had on mosquito trapping efficacy by increasing mosquito abundance estimates at communal roost sites based on the results from the artificial roosting experiment. We calculated the ratio of roost sizes of communal roosts and the number of American robins that would give off the same amount of CO_2_ as our artificial roost experiment. We then increased mosquito abundance estimates using this correction to calculate the amount of mosquitoes we would expect to catch at a communal roost site if nearby birds did not affect trapping success.

### Statistical analyses

We compared differences in host-seeking mosquito abundance at communal and non-communal roost sites, and variation in avian host roosting behavior using generalized mixed models formulated with the *glmer* and *lmer* functions in the lme4 package in R (v 2.15). For the mosquito abundance estimates we treated each individual mosquito trap and trapping period as random effects, and roost type (whether the collection site was used as an avian communal roost) as a fixed effect. In analyses of roosting height we used the identity of each individual bird as a random effect and modeled species as a fixed effect. Similarly, in analyzing variation in roost aggregation size among avian species the identity of individual birds was treated as a random effect and species and the interaction terms of species by Julian date and species by the square root of Julian date (to account for non-linearity) were included as fixed effects. We selected the best fitting mixed models using Akaike’s information criterion (AIC), and determined the significance of model terms by likelihood ratio tests on models with and without that predictor.

## Results

### Avian roosting and mosquito host-seeking behavior

**Figure 1:**
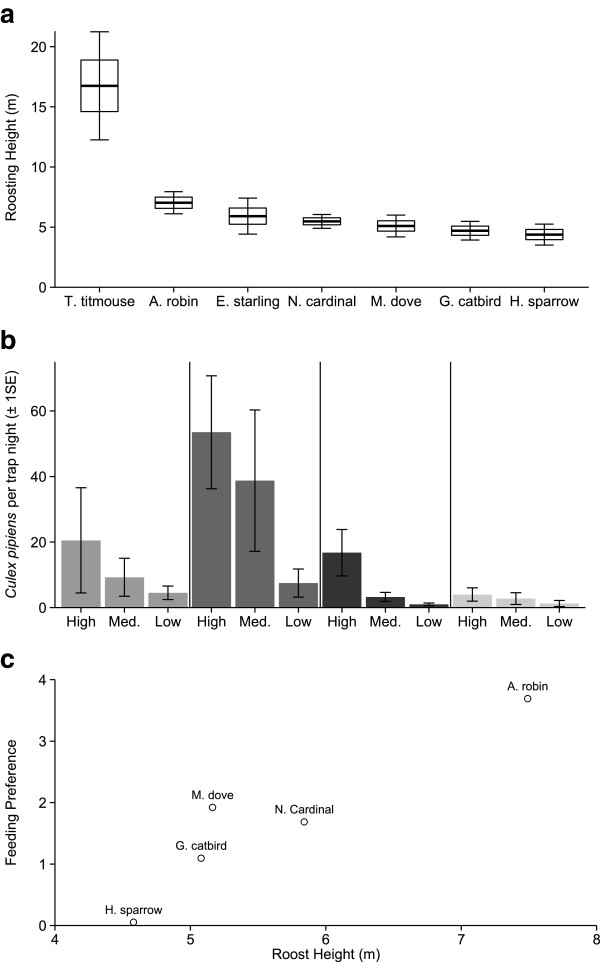
**Host roosting height, mosquito foraging height, and host-vector associations. a**. The roosting height of seven bird species in Maryland and Washington DC. Bold crossbars indicate the mean, boxes encompass ± 1 SE of the mean, and whisker lines represent 95% confidence bounds around the mean. Note that all but one E. starlings studied were adult birds whereas all other species studied were composed of juvenile birds only. **b**. Mean number of host-seeking *Cx. pipiens* mosquitoes collected per trap night (N = 48) at three traps along a single trap line at four trapping locations (separated by vertical bars) in Foggy Bottom, DC. **c**. Mosquito feeding preferences from an earlier study [28] plotted against average roost height for hatch year birds of each species.

We deployed 213 radio transmitters nearly four-fold more than previous studies on WNV host behavior [40, 41, 55], and quantified roosting behavior (roost height and group size) of 91 individuals of six species from July-September. Roosting height did not differ among sites (***χ***^2^ = 0.602, df =2, *P* = 0.74) but differed among species (Figure 1a; mixed-effects model with individual bird as a random effect, ***χ***^2^ = 36.59, df = 6, *P* < 0.001). Tufted titmice roosted highest in the canopy (data was primarily from at a single site near Rockville, MD), followed by American robins, and house sparrows roosted at the lowest height.

*Cx. pipiens* host-seeking mosquito abundance also increased with trap height (Figure 1b, generalized linear mixed-effects model with Poisson distribution and log-link, ***χ***^2^ = 24.51, df = 2, *P* < 0.001). Finally, feeding preferences of *Cx. pipiens* mosquitoes for each host increased with average roosting height at the same sites (Figure 1c; F_1,4_ = 22.21, *P* = 0.018).

**Figure 2:**
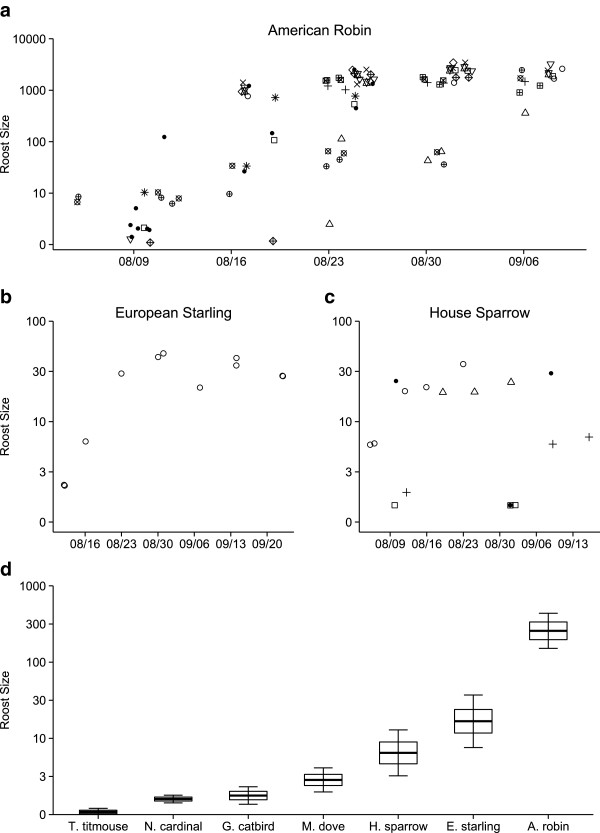
**Roost aggregation size for seven species of bird. a**. Temporal patterns of roost size for American robins, **b**. European starlings, and **c**. House sparrows. Roost sizes of individual American robins in panel 2a and house sparrows in panel 2c observed more than three times are represented by different open symbols. Closed circles show roost sizes for individuals observed less than three times. Points for American robins are jittered slightly to facilitate presentation. **d**. Mean roost size of for all species studied. Four species, Tufted titmouse, Northern cardinal, Gray catbird, and Mourning dove showed no significant variation over time; roost sizes for other species are estimated on the median date of the data in panels **(a-c)**. Bold crossbars indicate the mean, boxes encompass ± 1 SE of the mean, and whisker lines represent 95% confidence bounds around the mean.

Roost size, in numbers of birds, differed among species (Tables 1 and 2; species effect ***χ***^2^ = 107.89, df = 6, *P* < 0.001) and roost sizes of three of the seven species, American robins, European starlings, and house sparrows, increased from July to September (Figures 2a-c, Tables 1 and 2). In late fall, American robins occurred in the largest roosts, followed by European starlings and house sparrows. Mourning doves also roosted in groups, although aggregations for this species were much smaller than other communal roosting species (Figure 2d). Gray catbirds, northern cardinals, and tufted titmice often roosted individually or in pairs (Figure 2d).

**Table 1:** **Results of fitting a generalized linear mixed-effects model with a Poisson distribution and a log-link to analyze patterns of avian roost size over time for seven species of bird**

Predictor	Coefficient	SE	Z	P
**Intercept (A. Robin)**	-1075.1	15.9	-67.52	<0.001
**T. titmouse**	1454.6	602.8	2.41	0.016
**E. starling**	397.9	150.2	2.65	0.008
**G. catbird**	866.5	238.4	3.64	0.000
**H. sparrow**	566.7	171.3	3.31	0.001
**M. dove**	1888.5	301.2	6.27	<0.001
**N. cardinal**	978.8	134.1	7.3	<0.001
**Julian date (A. Robin)**	-4.4	0.1	-66.06	<0.001
**T. titmouse:Julian date**	6.0	2.6	2.32	0.021
**E. starling:Julian date**	1.7	0.6	2.72	0.007
**G. catbird:Julian date**	3.5	1.0	3.52	<0.001
**H. sparrow:Julian date**	2.3	0.7	3.11	0.002
**M. dove:Julian date**	7.9	1.3	6.06	<0.001
**N. cardinal:Julian date**	4.0	0.6	7.07	<0.001
**Julian date** ^**0.5**^ **(A. robin)**	137.5	2.1	66.97	<0.001
**T. titmouse:Julian date** ^**0.5**^	-186.7	78.8	-2.37	0.018
**E. starling:Julian date** ^**0.5**^	-51.5	19.1	-2.69	0.007
**G. catbird:Julian date** ^**0.5**^	-110.2	30.7	-3.59	<0.001
**H. sparrow:Julian date** ^**0.5**^	-72.2	22.4	-3.22	0.001
**M. dove:Julian date** ^**0.5**^	-244.3	39.6	-6.17	<0.001
**N. cardinal:Julian date** ^**0.5**^	-125.1	17.4	-7.2	<0.001

American robin was the reference level against which other effects were compared.

**Table 2:** **Comparison of models to explain variation among species and over time in roost size using Akaike’s Information Criterion (AIC), ΔAIC, Akaike weights (** ***ω***_***I***_ **)**

Model	AIC	ΔAIC	***ω***_***i***_	K
Species*Jdate + Species*Jdate^0.5^	16694	0	1	12
Species + Jdate	16965	271	0	9
Jdate + Jdate^0.5^	17135	441	0	3
Species*Jdate	22144	5450	0	10
Species + Jdate + Jdate^0.5^	22266	5572	0	10
Jdate	22418	5724	0	2
Species	41269	24575	0	8
Intercept	41373	24679	0	1

*denotes an interaction between two model parameters.

Parameters in the model include linear and nonlinear time components (Jdate is the Julian date or day of the year, with January 1 = 1). K is the number of parameters in the model.

**Figure 3:**
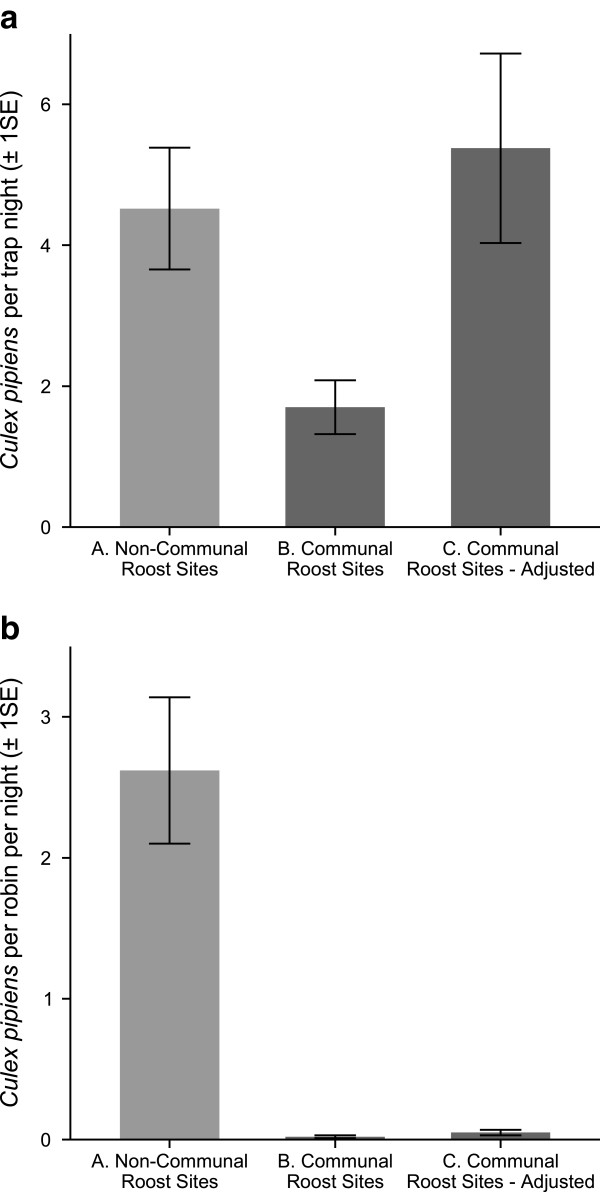
**Host-seeking mosquito abundance at communal and non-communal roost sites. a**. Mean number of host-seeking *Cx. pipiens* collected per trap night from August through September 2010 across (A) two non-communal roost sites and (B) two American robin communal roosts near Takoma Park, MD. Adjusted mosquito abundance estimates for communal roost sites (C) account for the effect hosts have on mosquito trapping efficacy. **b**. Per capita number of host seeking *Culex* mosquitoes per night at (A) two non-communal roost sites and (B) two American robin communal roosts near Takoma Park, MD. Adjusted mosquito:host ratio estimates for communal roost sites (C) account for the effect hosts have on mosquito trapping efficacy. Error bars encompass ± 1 SE of the mean.

Raw estimates of host-seeking mosquito abundance varied among two communal and two non-communal roost sites (generalized linear mixed-effects model with log-link; site effect: ***χ***^2^ = 9.85, df = 3, *P* = 0.02) but, surprisingly, traps at American robin communal roost sites caught significantly fewer mosquitoes than traps at non-communal roosting areas (Figure 3a; generalized linear mixed-effects model with log-link, ***χ***^2^ = 4.03, df = 1, *P* = 0.04). The vector:host ratios at communal roost sites using these raw data on host seeking mosquito abundance were 0.0011 ± 0.0004 mosquitoes per American robin per night at the two communal roost sites and 2.62 ± 0.52 mosquitoes per American robin per trap night at non-communal roost sites, or more than two-thousand fold higher (Figure 3b).

### Trapping bias - effect of hosts near traps

**Figure 4:**
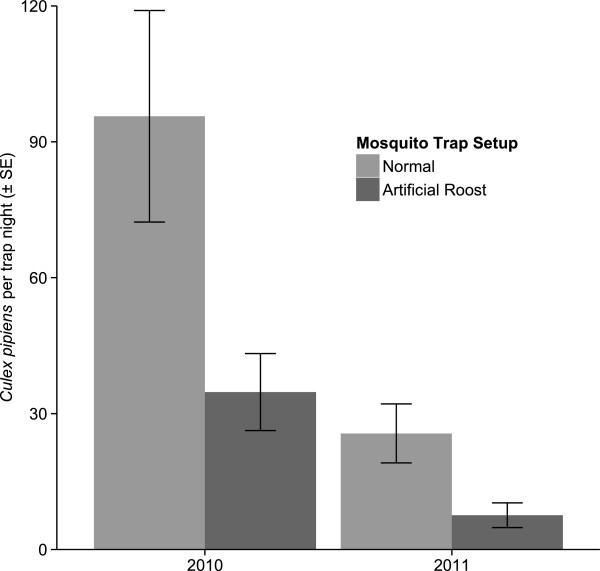
**Mean number of host-seeking** ***Cx. pipiens*** **trapped at normal trap setup locations and at locations with traps surrounded by four coolers full of dry ice to simulate artificial roosts.**

Placing additional coolers of CO_2_ near mosquito traps to simulate birds roosting nearby reduced the number of mosquitoes caught by 57% ± 12% (Figure 4; generalized linear mixed-effects model with a Poisson distribution and a log-link, ***χ***^2^ = 298.04, df = 1, *P* < 0.001). We used this and the approximate number of birds that would produce the same amount of CO_2_ (372.4 ± 24.8; see Methods), to adjust for the effect of additional hosts near mosquito traps at communal roost sites. This adjustment increased mosquito abundance estimates from 1.23 ± 0.4 to 3.6 ± 1.1 mosquitoes per trap night at communal roosts. With these adjusted estimates, there was no longer a significant difference in mosquito abundance between communal roosts and non-communal roosting areas (Figure 4; generalized linear mixed-effects model with a Poisson distribution and a log-link, ***χ***^2^ = 0.0372, df = 1, *P* = 0.85). However, the estimated vector:host ratios using adjusted mosquito abundance at roosts were still more than 850 times lower at communal roost sites (0.003 ± 0.001 mosquitoes per American robin) than at sites not used for communal roosting (2.62 ± 0.52 mosquitoes per American robin, Figure 3b).

## Discussion

Our results suggest two general aspects of host behavior, sociality and fine-scale habitat use, which have received relatively little research effort [40, 41, 55], influence patterns of vector-host interactions, and could thereby alter both transmission of and exposure to vector-borne pathogens. Three species, American robins, European starlings, and house sparrows, all formed communal roosts as mosquito abundance increased and infection prevalence with WNV in this region and others subsequently decreased [29, 30, 40, 41, 56]. However, although large aggregations of birds produce substantial amounts of CO_2_ that might be expected to attract many host-seeking mosquitoes, per capita mosquito abundance at roost sites was two orders of magnitude lower than at non-communal roost sites. Even though we were not able to fully adjust our estimates of mosquito abundance for the distraction effect of birds roosting near mosquito traps (additional coolers of dry ice did not capture or satiate foraging mosquitoes, and did not include other odors emitted by birds), the enormous reduction in vector:host ratio suggests that sociality in these communally roosting species substantially reduces their contact with biting mosquito vectors.

Reduction in average contact rates with biting vectors would reduce exposure to vector-borne pathogens. This would represent an additional fitness benefit conferred from communal roosting that complements other better studied effects such as protection from predators and centers for sharing foraging information [57]. Communal roosting behavior in American robins, European starlings and house sparrows occurred well before the arrival of WNV to North America and it is unclear how important the influence of other arboviruses such as St. Louis encephalitis virus has been in the evolution of communal roosting behavior. However, the introduction of WNV could have altered the selective pressure and timing of selection on communal roosting. The density of WNV-infected mosquitoes in the study area peaks in late July – early August [29], when communal roosts begin forming at our sites and elsewhere [40, 41]. Given that approximately 12% of American robins infected with WNV die from infection [58], the selective pressure of WNV on American robins to form communal roosts could be non-trivial [59].

Our results suggest that habitat use, - specifically foraging and roosting height, by vectors and hosts, respectively, also plays a significant role in host-vector contact. As in previous studies [36–39], we found that host-seeking *Culex* abundance was nearly four times higher at mid-level traps and six times higher at high-level traps compared to traps placed 1.5 m from the ground. This suggests that mosquito-biting rates would be lowest on host species roosting lower in the canopy, and we found preliminary evidence of a correlation between mosquito feeding preference and avian roosting height. Higher host-seeking mosquito abundance in the canopy could result from CO_2_ plumes from both traps and birds in the canopy spreading further than plumes from birds or traps at lower heights. House sparrows, which roost quite low (often in shrubs) may experience reduced mosquito contact rates due to mosquito foraging heights which could partially provide an explanation for the very low feeding preferences or host utilization on this species in many studies [27, 28, 30]. Further work on additional species and in other regions will determine whether roosting height is a consistent predictor of mosquito feeding preference.

Previous work has shown that feeding of *Cx. pipiens* on a preferred avian host, American robins, decreases in the fall [27, 28, 30, 44] and this decrease has been associated with an increase in human feeding and human cases of WNV in one region [29]. This feeding shift was hypothesized to result from the late-summer dispersal of American robins following breeding. Our results suggest that the movement of American robins to communal roosts during this period might make them less available to host-seeking mosquitoes in urban and residential areas away from roosts and account for the reduced feeding on this species. The period when birds start forming communal roosts also coincides with a decrease in WNV infection prevalence in mosquitoes [29]. A decrease in transmission at this time would be expected based our results that communal roosting reduces vector-host ratios on the most important species in WNV amplification in this and other regions, American robins [26–30, 44].

Our findings raise questions about previous assertions that large aggregations of American robins increase WNV transmission [40]. While Diuk-Wasser *et al.* found higher WNV prevalence in mosquitoes at communal American robin roost sites compared to non-roost sites, they found no difference in total *Culex* mosquito abundance among roost and non-roost sites, and the ratio of per capita abundance of *Culex* mosquitoes at roost sites and non-roost sites was similar to what we found (<800 fold lower at the communal roost). This raises the question of how communal roosting could have increased transmission at roost sites if per capita mosquito abundance was much lower. Two possibilities are: 1) that other non-roost related factors differed among sites (roost sites are not randomly selected by birds or scientists) and 2) biting at roosts could be focused on a very small subset of the birds at the communal roost resulting in a higher effective vector:host ratio than one considering all birds in the roost. Additional work examining WNV transmission ecology at a larger number of communal roost sites compared to sites not used for communal roosting would help answer this question, especially with mosquito trapping and testing for WNV occurring before roost formation. These studies should also account for the biases we have identified from mosquitoes being differentially attracted to traps at communal roost sites.

The distribution of mosquitoes attempting to feed on individual birds within a roost also likely influences host defensive behavior and feeding success [32–34]. As a result, communal roosting by birds likely disrupts the optimal distribution of mosquitoes feeding on each bird, because too many mosquitoes per bird are feeding at non-communal roost sites, likely leading to increased host defensive behavior and reduced mosquito feeding success [14].

## Conclusions

In summary, our results demonstrate how host aggregation and habitat use and vector searching behavior influence contacts between vectors and hosts. Differential habitat use by different host species and spatial heterogeneity in vector host-seeking behavior partly explains why vectors rarely feed on hosts in proportion to their abundance. Our results also demonstrate the importance of quantifying the per capita rate of contact between vectors and communally roosting hosts and underscore the need to consider the spatial and among-individual heterogeneities in the disease ecology landscape.

## References

[1] Altizer S, Nunn CL, Thrall PH, Gittleman JL, Antonovics J, Cunningham AA, Dobson AP, Ezenwa V, Jones KE, Pedersen AB, Poss M, Pulliam JRC (2003). Social organization and parasite risk in mammals: Integrating theory and empirical studies. Annu Rev Ecol Evol Syst.

[2] Johnson CK, Tinker MT, Estes JA, Conrad PA, Staedler M, Miller MA, Jessup DA, Mazet JAK (2009). Prey choice and habitat use drive sea otter pathogen exposure in a resource-limited coastal system. Proc Natl Acad Sci U S A.

[3] Paull SH, Song SJ, McClure KM, Sackett LC, Kilpatrick AM, Johnson PTJ (2012). From superspreaders to disease hotspots: linking transmission across hosts and space. Front Ecol Environ.

[4] Venesky MD, Kerby JL, Storfer A, Parris MJ (2011). Can differences in host behavior drive patterns of disease prevalence in tadpoles?. Plos One.

[5] Moore CG (2008). Interdisciplinary research in the ecology of vector-borne diseases: opportunities and needs. J Vector Ecol.

[6] Farajollahi A, Fonseca DM, Kramer LD, Kilpatrick AM (2011). Bird-biting mosquitoes and human disease: a review of the role of *Culex pipiens* complex mosquitoes in epidemiology. Inf Gen Evol.

[7] Cohen JE, Gurtler RE (2001). Modeling household transmission of American trypanosomiasis. Science.

[8] Martens P, Hall L (2000). Malaria on the move: human population movement and malaria transmission. Emerg Infect Dis.

[9] Stoddard ST, Morrison AC, Vazquez-Prokopec GM, Soldan VP, Kochel TJ, Kitron U, Elder JP, Scott TW (2009). The role of human movement in the transmission of vector-borne pathogens. PLoS Negl Trop Dis.

[10] Kilpatrick AM, Randolph SE (2012). Drivers, dynamics, and control of emerging vector-borne zoonotic diseases. Lancet.

[11] Kilpatrick AM (2011). Globalization, land use, and the invasion of West Nile virus. Science.

[12] Takken W, Verhulst NO (2013). Host preferences of blood-feeding mosquitoes. Annu Rev Entomol.

[13] Ngom EM, Ndione JA, Ba Y, Konate L, Faye O, Diallo M, Dia I (2013). Spatio-temporal analysis of host preferences and feeding patterns of malaria vectors in the sylvo-pastoral area of Senegal: impact of landscape classes. Paras Vec.

[14] Kelly DW, Thompson CE (2000). Epidemiology and optimal foraging: modelling the ideal free distribution of insect vectors. Parasitology.

[15] Hamilton WD (1971). Geometry for the selfish herd. J Theor Biol.

[16] Mooring MS, Hart BL (1992). Animal grouping for protection from parasites: selfish herd and encounter-dilution effects. Behaviour.

[17] Anderson J, Nilssen A (1998). Do reindeer aggregate on snow patches to reduce harassment by parasitic flies or to thermoregulate?. Rangifer.

[18] Helle T, Aspi J (1983). Does herd formation reduce insect harassment among reindeer? A field experiment with animal traps. Acta Zool Fenn.

[19] Brown CR, Sethi RA (2002). Mosquito abundance is correlated with Cliff Swallow (*Petrochelidon pyrrhonota*) colony size. J Med Entomol.

[20] Poulin R (1991). Group-living and infestation by ectoparasites in Passerines. Condor.

[21] Cote IM, Poulin R (1995). Parasitism and group size in social animals: a meta-analysis. Behav Ecol.

[22] Kilpatrick AM, Kramer LD, Campbell S, Alleyne EO, Dobson AP, Daszak P (2005). West Nile virus risk assessment and the bridge vector paradigm. Emerg Infect Dis.

[23] Turell MJ, Sardelis MR, O'Guinn ML, Dohm DJ, Mackenzie J, Barrett A, Deubel V (2002). Potential vectors of West Nile virus in North America. Japanese Encephalitis and West Nile Viruses.

[24] Hamer GL, Kitron UD, Brawn JD, Loss SR, Ruiz MO, Goldberg TL, Walker ED (2008). *Culex pipiens* (Diptera: Culicidae): a bridge vector of West Nile virus to humans. J Med Entomol.

[25] Ciota AT, Chin PA, Kramer LD (2013). The effect of hybridization of *Culex pipiens* complex mosquitoes on transmission of West Nile virus. Paras Vec.

[26] Hamer GL, Chaves LF, Anderson TK, Kitron UD, Brawn JD, Ruiz MO, Loss SR, Walker ED, Goldberg TL (2011). Fine-scale variation in vector host use and force of infection drive localized patterns of West Nile virus transmission. Plos One.

[27] Hamer GL, Kitron UD, Goldberg TL, Brawn JD, Loss SR, Ruiz MO, Hayes DB, Walker ED (2009). Host selection by *Culex pipiens* mosquitoes and West Nile virus amplification. Am J Trop Med Hyg.

[28] Kilpatrick AM, Daszak P, Jones MJ, Marra PP, Kramer LD (2006). Host heterogeneity dominates West Nile virus transmission. Proc R Soc B.

[29] Kilpatrick AM, Kramer LD, Jones MJ, Marra PP, Daszak P (2006). West Nile virus epidemics in North America are driven by shifts in mosquito feeding behavior. PLoS Biol.

[30] Kent R, Juliusson L, Weissmann M, Evans S, Komar N (2009). Seasonal blood feeding behavior of *Culex tarsalis* (Diptera: Culicidae) in Weld County, Colorado, 2007. J Med Entomol.

[31] Kilpatrick AM, LaDeau SL, Marra PP (2007). Ecology of West Nile virus transmission and its impact on birds in the western hemisphere. Auk.

[32] Anderson RA, Brust RA (1997). Interrupted blood feeding by Culex (Diptera: Culicidae) in relation to individual host tolerance to mosquito attack. J Med Entomol.

[33] Edman J, Webber L, Kale H (1972). Effect of mosquito density of interrelationship of host behavior and mosquito feeding success. Am J Trop Med Hyg.

[34] Hodgson JC, Spielman A, Komar N, Krahforst CF, Wallace GT, Pollack RJ (2001). Interrupted blood-feeding by *Culiseta melanura* (Diptera: Culicidae) on European starlings. J Med Entomol.

[35] Scott TW, Lorenz LH, Edman JD (1990). Effects of house sparrow age and arbovirus infection on attraction of mosquitos. J Med Entomol.

[36] Anderson JF, Andreadis TG, Main AJ, Ferrandino FJ, Vossbrinck CR (2006). West Nile virus from female and male mosquitoes (Diptera : Culicidae) in subterranean, ground, and canopy habitats in Connecticut. J Med Entomol.

[37] Deegan CS, Burns JE, Huguenin M, Steinhaus EY, Panella NA, Beckett S, Komar N (2005). Sentinel pigeon surveillance for West Nile virus by using lard-can traps at differing elevations and canopy cover classes. J Med Entomol.

[38] Drummond CL, Drobnack J, Backenson PB, Ebel GD, Kramer LD (2006). Impact of trap elevation on estimates of abundance, parity rates, and body size of *Culex pipiens* and *Culex restuans* (Diptera : Culicidae). J Med Entomol.

[39] Savage HM, Anderson M, Gordon E, McMillen L, Colton L, Delorey M, Sutherland G, Aspen S, Charnetzky D, Burkhalter K, Godsey M (2008). Host-seeking heights, host-seeking activity patterns, and West Nile virus infection rates for members of the *Culex pipiens* complex at different habitat types within the hybrid zone, Shelby County, TN, 2002 (Diptera: Culicidae). J Med Entomol.

[40] Diuk-Wasser MA, Molaei G, Simpson JE, Folsom-O'Keefe CM, Armstrong PM, Andreadis TG (2010). Avian communal roosts as amplification foci for West Nile virus in urban areas in northeastern United States. Am J Trop Med Hyg.

[41] Benson TJ, Ward MP, Lampman RL, Raim A, Weatherhead PJ (2012). Implications of spatial patterns of roosting and movements of American robins for West Nile virus transmission. Vector-Borne Zoonotic Dis.

[42] MacDonald G (1957). The Epidemiology and Control of Malaria.

[43] Ross R (1910). The prevention of malaria.

[44] Savage HM, Aggarwal D, Apperson CS, Katholi CR, Gordon E, Hassan HK, Anderson M, Charnetzky D, McMillen L, Unnasch EA, Unnasch TR (2007). Host choice and West Nile virus infection rates in blood fed mosquitoes, including members of the *Culex pipiens* complex, from Memphis and Shelby County, Tennessee 2002–2003. Vector-Borne Zoonotic Dis.

[45] Bernard KA, Maffei JG, Jones SA, Kauffman EB, Ebel GD, Dupuis AP, Ngo KA, Nicholas DC, Young DM, Shi PY, Kulasekera VL, Edison M, White DJ, Stone WB, Kramer LD, NY State West Nile Virus Surveillance Team (2001). West Nile virus infection in birds and mosquitoes, New York State, 2000. Emerg Infect Dis.

[46] Cochran W, SD S (1980). Wildlife Telemetry. The Wildlife Management Techniques Manual.

[47] Hamer GL, Walker ED, Brawn JD, Loss SR, Ruiz MO, Goldberg TL, Schotthoefer AM, Brown WM, Wheeler E, Kitron UD (2008). Rapid amplification of West Nile virus: the role of hatch-year birds. Vector-Borne Zoonotic Dis.

[48] Rappole JH, Tipton AR (1991). New harness design for attachment of radio transmitters to small passerines. J Field Ornithology.

[49] Wanless S, Harris MP, Morris JA (1991). Foraging range and feeding locations of shags *Phalacrocorax aristotelis* during chick rearing. Ibis.

[50] Smith JAM, Reitsma LR, Rockwood LL, Marra PP (2008). Roosting behavior of a Neotropical migrant songbird, the northern waterthrush *Seiurus noveboracensis*, during the non-breeding season. J Avian Biol.

[51] Gillies M, Wilkes T (1970). The range of attraction of single baits for some West African mosquitoes. Bull Entomol Res.

[52] Darsie RFJ, Ward RA (1981). Identification and geographic distribution of mosquitoes of North America, north of Mexico. Mosq Syst Suppl.

[53] Crabtree MB, Savage HM, Miller BR (1995). Development of a species-diagnostic polymerase chain reaction assay for the identification of *Culex* vectors of St. Louis encephalitis virus based on interspecies sequence variation in ribosomal DNA spacers. Am J Trop Med Hyg.

[54] Nagy KA, Girard IA, Brown TK (1999). Energetics of free-ranging mammals, reptiles and birds. Annu Rev Nutr.

[55] Ward MP, Raim A, Yaremych-Hamer S, Lampman R, Novak RJ (2006). Does the roosting behavior of birds affect transmission dynamics of West Nile virus?. Am J Trop Med Hyg.

[56] Morrison D, Caccamise D (1990). Comparison of roost use by three species of communal roostmates. Condor.

[57] Ward P, Zahavi A (1973). The importance of certain assemblages of birds as “information-centres” for food-finding. Ibis.

[58] VanDalen KK, Hall JS, Clark L, McLean RG, Smeraski C (2013). West Nile virus infection in American robins: new insights on dose response. Plos One.

[59] Kilpatrick AM (2006). Facilitating the evolution of resistance to avian malaria in Hawaiian birds. Biol Conserv.

